# WS_2_ Nanorod as a Remarkable Acetone Sensor for Monitoring Work/Public Places

**DOI:** 10.3390/s22228609

**Published:** 2022-11-08

**Authors:** Rajneesh Kumar Mishra, Vipin Kumar, Le Gia Trung, Gyu Jin Choi, Jeong Won Ryu, Sagar M. Mane, Jae Cheol Shin, Pushpendra Kumar, Seung Hee Lee, Jin Seog Gwag

**Affiliations:** 1 Department of Physics, Yeungnam University, Gyeongsan 38541, Korea; 2 Division of Electronics and Electrical Engineering, Seoul Campus, Dongguk University, Seoul 04620, Korea; 3 Department of Physics, Manipal University Jaipur, Jaipur 303007, India; 4 Department of Nanoconvergence Engineering, Jeonbuk National University, Jeonju 54896, Korea; 5 Department of Polymer Nano-Science and Technology, Jeonbuk National University, Jeonju 54896, Korea

**Keywords:** WS_2_ nanorods, gas sensors, acetone sensing, selective nature, durability, acetone sensing mechanism

## Abstract

Here, we report the synthesis of the WS_2_ nanorods (NRs) using an eco-friendly and facile hydrothermal method for an acetone-sensing application. This study explores the acetone gas-sensing characteristics of the WS_2_ nanorod sensor for 5, 10, and 15 ppm concentrations at 25 °C, 50 °C, 75 °C, and 100 °C. The WS_2_ nanorod sensor shows the highest sensitivity of 94.5% at 100 °C for the 15 ppm acetone concentration. The WS_2_ nanorod sensor also reveals the outstanding selectivity of acetone compared to other gases, such as ammonia, ethanol, acetaldehyde, methanol, and xylene at 100 °C with a 15 ppm concentration. The estimated selectivity coefficient indicates that the selectivity of the WS_2_ nanorod acetone sensor is 7.1, 4.5, 3.7, 2.9, and 2.0 times higher than xylene, acetaldehyde, ammonia, methanol, and ethanol, respectively. In addition, the WS_2_ nanorod sensor also divulges remarkable stability of 98.5% during the 20 days of study. Therefore, it is concluded that the WS_2_ nanorod can be an excellent nanomaterial for developing acetone sensors for monitoring work/public places.

## 1. Introduction

The rapidly increasing industrial evolutions in the fields of agriculture, automobiles, biomedical, and food packaging have introduced significant concerns about environmental monitoring technologies, leading to the development of reliable and durable gas sensors [1]. The human exhaled breath contains numerous types of gases, such as ketones, nitric oxide, aldehydes, volatile organic compounds, acids, and hydrogen sulfide [2,3]. Therefore, exhaled human breath is a significant and rousing issue from the outlook of biomedical applications to inspect different diseases. Interestingly, the exhaled human breath contains nearly 870 volatile organic compound types, indicating exclusive evidence regarding metabolic disorders [4]. Therefore, studying exhaled human breath can provide insights into crucial results of humans’ normal or abnormal metabolic states arising from psychological stress [5]. Acetone molecules have been considered hazardous to human health and the environment. Acetone is a member of a family of volatile organic compounds that can influence the human nervous system and other organs under excessive exposure to concentrations of nearly 173 ppm [6]. Acetone is a vital aspect of the human metabolic system and can be examined through blood, breath, and urine [7,8]. It has been found to be a precise biomarker to recognize individuals with diabetes type-I due to the presence of high acetone vapor in the exhaled breath compared to healthy humans [9]. Various sensors, such as electrochemical, colorimetric, and resistive chemical gas, have been studied to detect acetone [10,11,12]. The colorimetric sensor provides low accuracy and requires additional setups such as airbags and pumps, which makes it very expensive [11]. However, the resistive chemical gas sensor offers high sensitivity, stability, fast responses, portability, and recovery, which makes it a cheap gas-sensing setup with low-cost sensor fabrication [12].

Among various transition metal dichalcogenides (TMDs), tungsten disulfide (WS_2_) is considered the most prominent two-dimensional (2D) nanomaterial for developing novel applications [13]. 2D layered nanomaterials have been extensively examined because of their remarkable properties, such as physical, optical, electronic, and mechanical, which can stimulate their performance in various applications [14]. WS_2_, a member of the layered hexagonal family, is a promising nanomaterial with 0.62 nm interlayer spacing composed of covalently bonded S-W-S atoms, where each layer is weakly bonded by Van der Walls forces [15,16]. WS_2_ has no dangling bonds, which makes it extremely stable and non-reactive. It can absorb nearly 10% of the incident light due to its high absorption coefficient of 1.5 × 10^6^ cm^−1^ [17]. It offers the desired engineering in the optical bandgap with high photoluminescence yields due to quantum confinements [18]. Interestingly, it exhibits an exclusive property of an engineering optical bandgap from an indirect optical bandgap of 1.4 eV (bulk) to a direct optical bandgap of 2.1 eV (monolayer), which provides a spin–orbit solid interaction [18,19]. Therefore, the WS_2_ nanostructure has been studied in numerous types of applications, such as monolayer-based field-effect transistors [20], solar cells [21], monolayer-based light-emitting diodes [22], gas sensors [23], neuromorphic devices [24], biosensors [25], supercapacitors [26], lithium-ion batteries [27], and electrocatalytic [28] and photocatalytic water splitting [29]. WS_2_ has been prepared using various methods, such as hydrothermal [30], solvothermal [31], chemical vapor deposition (CVD) [32], hot injection [33], thermal evaporation [34], and DC sputtering [35]. WS_2_ has been explored in various types of morphologies, such as quantum dots [36], heterostructures [37], nanowire-nanoflake [38], nanorods [39], nanoflowers [40], and nanosheets [41]. 

Recently, various morphologies of WS_2_ have been investigated for the detection of different types of gas. Liu et al. discussed the acetone gas-sensing behavior of WS_2_/WO_3_ heterojunctions [42]. The WS_2_/WO_3_-4 heterojunctions sensor offers reasonable sensitivity for acetone at a concentration of 20 ppm at 150 °C, selectivity in the presence of various hazardous gases, and stability for one month at a 150 °C working temperature for 20 ppm. Tang et al. inspected the NO_2_ gas-sensing performance of the WS_2_/IGZO p-n heterojunction sensor [23]. It shows a response of 230% for 5 ppm NO_2_ gas and 18,170% for 300 ppm NO_2_ gas. It also suggests that the recovery percentage increases with increasing the gas concentration. Kim et al. studied the WS_2_ nanosheet-based carbon monoxide gas sensor [43]. It depicts the CO response of 3 for 50 ppm concentration and the selective response of 3.75 for 50 ppm CO. It shows the response time of 339 s and recovery time of 567 s for 50 ppm CO. Ahmadvand et al. reported the ethanol sensor using the hybrid structure of the WS_2_ and graphene oxide nanoribbons (WS_2_/GONRs) [44]. It is observed that the WS_2_/GONRs show responses of 13.5 for 1 ppm and 438.5 for 21 ppm concentrations of ethanol at room temperature. Guang et al. explored ammonia sensing characteristics using the Au-coated WS_2_ [45]. It shows a good gas response of 452% for 10 ppm of ammonia at room temperature. It also proposes a response time of 96 and a recovery time of 76 at room temperature for 10 ppm ammonia. Asres et al. investigated the H_2_S sensing properties using the WS_2_ sensor [46]. It shows that the WS_2_ sensor behaves as a robust gas sensor for a high H_2_S response. However, very limited reports exist for acetone sensing using the WS_2_ sensing elements. Therefore, it is concluded that acetone detection using WS_2_ sensors needs more attention from researchers to develop an extremely selective, responsive, and long-life gas sensor. 

In this study, the WS_2_ nanorods were prepared using the hydrothermal method for the acetone-sensing application. The WS_2_ nanorod sensor displays outstanding acetone-sensing properties for 15 ppm concentrations at an operating temperature of 100 °C. It is observed that the WS_2_ nanorod sensor shows a rapid response and fast recovery time. Furthermore, the WS_2_ nanorod sensor displays excellent acetone selectivity compared to other test gases. Moreover, the WS_2_ nanorod sensor reveals outstanding stability during its long-term use. Therefore, it is concluded that these unique properties make it a remarkable acetone sensor for future applications in work/public places. 

## 2. Materials and Methods

### 2.1. Materials Synthesis

The tungsten(IV) chloride, thioacetamide, ethanol, polyvinylidene fluoride (PVDF), hexamethyldisilazane (HMDS), xylene, methanol, N-Methyl-2-pyrrolidone (NMP), ammonia, and acetaldehyde were purchased from Sigma-Aldrich (St. Louis, MO, USA), which were utilized for the synthesis of WS_2_ nanorods as received.

The WS_2_ nanorods were prepared using a facile and eco-friendly hydrothermal process. In the synthesis process, 0.1904 g of the tungsten(IV) chloride compound was mixed in 80 mL of deionized (DI) water under dynamic stirring to achieve a good mixture. After that, 0.1803 g of thioacetamide was put into the prepared mixture solution using magnetic stirring to prepare a well-mixed solution. Furthermore, we added 1 mL of hexamethyldisilazane (HMDS) as a surfactant to control the morphology of the desired final product. Moreover, this prepared solution of tungsten(IV) chloride and thioacetamide was transferred to the 100 mL Teflon-lined autoclave. Further, this solution-filled autoclave was put into an air oven at 180 °C for twenty-four hours. Finally, the as-prepared nanomaterials were rinsed with ethanol and DI water. Further, it was dried at 70 °C for 10 h in a vacuum oven and processed with heat treatment at 200 °C for 3 h under a vacuum. 

### 2.2. Materials Characterizations

Transmission electron microscopy (TEM) (JEOL JEM 2100F, JEOL Ltd., Tokyo, Japan) was utilized to explore the structural, morphological, lattice spacings, and lattice planes of WS_2_ nanorods. In addition, the atomic-resolution high-angle annular dark-field (HAADF) and electron energy loss spectroscopy (EELS) were investigated using a JEOL, JEM-2100F, JEOL Ltd., Tokyo, Japan, to study the elemental color mapping of WS_2_ nanorods. 

### 2.3. WS_2_ NRs-Based Sensor Fabrication and Measurements

The following process was used to fabricate the WS_2_ nanorods-based acetone sensor: The first binder solution of 0.5 g of polyvinylidene fluoride (PVDF) was prepared using solvent N-Methyl-2-pyrrolidone (NMP) in a drop-wise manner. After that, we slowly added WS_2_ nanorod powder to the binder solution and mixed it well to obtain the desired solution for sensor fabrication. The WS_2_ nanorod gluey solution was coated on the glass substrate using drop-casting and dried slowly at 40 °C. Further, the silver paste was used to make contact on both sides of the film (deposited on glass) for the electrical connection. The gas-sensing measurements were conducted using the Keithley-2100 multimeter; however, the Motwane-454 multimeter was used to maintain the temperature inside the test chamber. The acetone gas-sensing measurements were investigated at 25 °C–100 °C for 5 ppm, 10 ppm, and 15 ppm concentrations. The acetone concentrations were injected into the test gas chamber using a Hamilton micro-syringe. In addition, the volume of the acetone concentrations (C, ppm) was estimated using Equation (1) [47]:

(1) $$C\;\left(ppm\right)=\frac{22.4\times T\times V_{l}\times \rho }{V\times M}\times 1000$$

where *C* (*ppm*) is the desired acetone concentration, *ρ* (g L^−1^) is the liquid acetone density, *V_l_* (μL) is the volume of the liquid acetone, *M* (g mol^−1^) is the molecular weight of the acetone, *T* (°C) is the working temperature of acetone sensing, and *V* is the acetone gas test chamber.

## 3. Results and Discussion

### 3.1. Morphological, Structural, and Elemental Study

**Figure 1:**
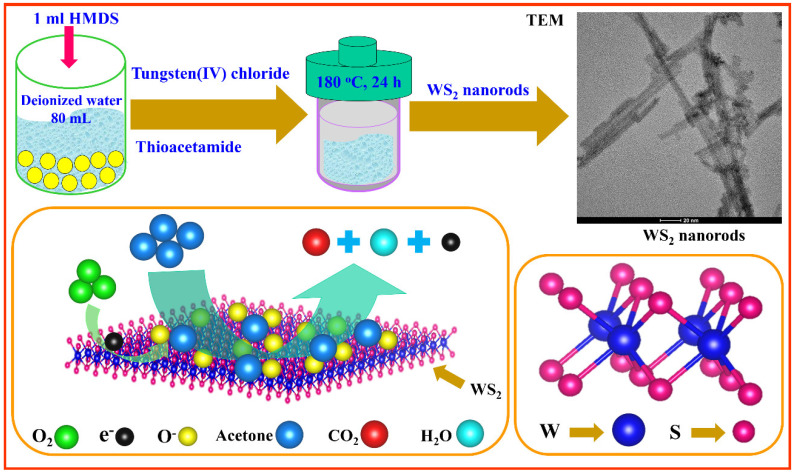
Concept of the WS_2_ nanorod synthesis method. Crystallographic presentation of WS_2_ crystal structure. Graphic of the acetone-sensing mechanism of the WS_2_ sensor. Visualization and interactions of molecules and electrons during acetone sensing of WS_2_ sensor.

Figure 1 shows the schematic depiction of synthesis, the crystal structure, and the acetone-sensing properties of the WS_2_ nanorods. It depicts the solution preparation of tungsten and thioacetamide with the HMDS surfactant. It also reveals the hydrothermal reaction conditions at 180 °C for 24 h. Furthermore, it verifies the successful synthesis of WS_2_ nanorods via TEM images. It shows the crystallographic illustration of the WS_2_ crystal structure and the visualization of the acetone-sensing mechanism. The detailed synthesis procedure was discussed in the material synthesis section. The detailed synthesis procedure was discussed in the acetone-sensing mechanism section. 

**Figure 2:**
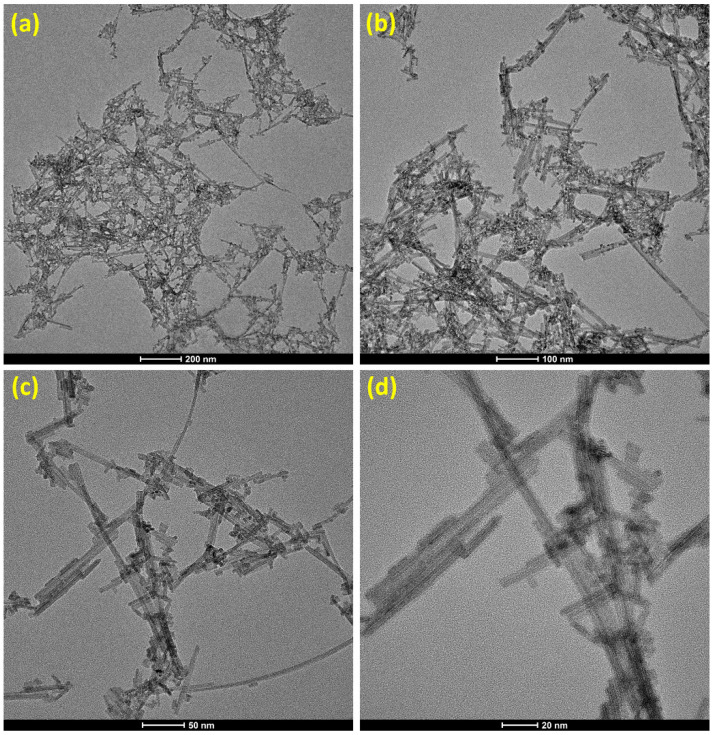
(**a**–**d**) TEM images of the WS_2_ nanorods at the different scales.

Figure 2a–d display the morphology of the WS_2_ nanorods using the TEM at different scale bars. Figure 2a,b depict the agglomerations of the WS_2_ nanorods with various sizes and lengths. It seems to be overlapped, crossed WS_2_ nanorods, which can improve oxygen molecules’ conduction mechanism and interactions with the nanorods and gas-sensing properties. Figure 2c displays the WS_2_ nanorods whose length varies from nearly 20 nm to 200 nm. It also elucidates the WS_2_ nanorods whose widths fluctuate from 3 nm to 6 nm. Figure 2d shows several attached WS_2_ nanorods, forming various intersections between nanorods. It also reveals a thickness of 3 nm to 6 nm and a length of 20 nm to 200 nm of WS_2_ nanorods. It seems to develop several solid connections/attachments between the nanorods, which offer a large surface area, more adsorption, and chemisorption of atmospheric oxygen and gas molecules. 

**Figure 3:**
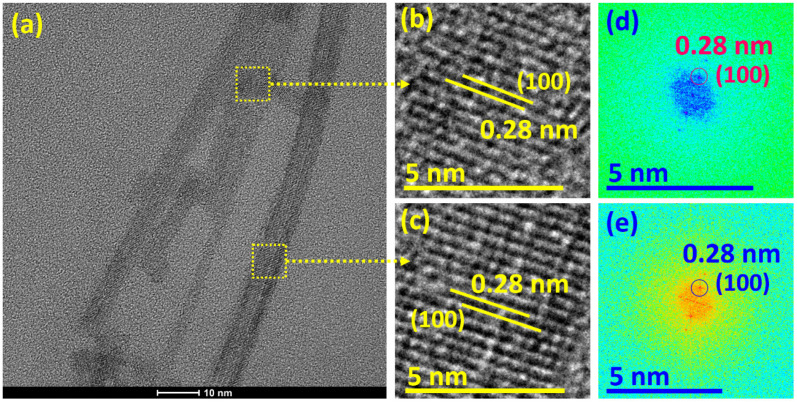
(**a**) HRTEM image, (**b**,**c**) magnified HRTEM images, and corresponding (**d**,**e**) FFT patterns of the WS_2_ nanorods.

Figure 3a–e show the HRTEM and fast Fourier transform (FFT) patterns to explore the crystal structure, lattice spacing, and lattice planes of the WS_2_ nanorods. Figure 3a displays the HRTEM image of the WS_2_ nanorods with 2 to 5 nm thickness, and the length varies from 20 nm to 100 nm. Figure 3b,c reveal the magnified HRTEM images of the WS_2_ nanorods, indicating lattice spacings of 0.28 nm corresponding to the (100) lattice plane. Further, Figure 3d,e exhibit the FFT patterns of the WS_2_ nanorods to study the lattice plane and spacing, signifying the growth of the (100) lattice plane corresponding to the lattice spacing of 0.28 nm. The FFT pattern justifies the lattice spacing results, as shown in Figure 3a. The literature reports discussed similar HRTEM and FFT results of WS_2_ nanostructures [48,49].

**Figure 4:**
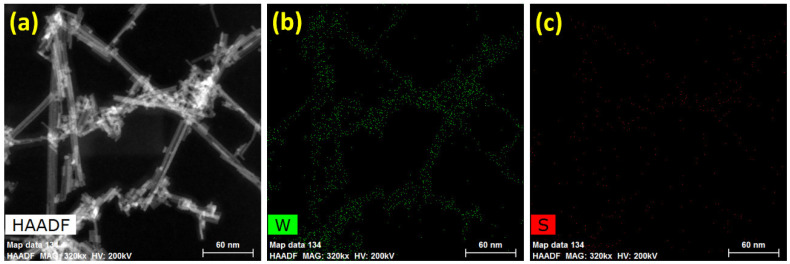
(**a**) HAADF image and corresponding color mapping of (**b**) tungsten and (**c**) sulfur elements of the WS_2_ nanorods.

In addition, the elemental information of the WS_2_ nanorods was studied using the HAADF image consistent with the color mapping of tungsten and sulfur elements. Figure 4a–c unveil the dark-field TEM (HAADF) image and corresponding elemental mapping of tungsten and sulfur elements of the WS_2_ nanorods. Figure 4a divulges the HAADF image of the WS_2_ nanorods to examine the elements’ composition and presence in the desired area. It also shows short to long WS_2_ nanorods with thin diameters. Figure 4b,c reveal the color mapping of tungsten and sulfur elements of the WS_2_ nanorods from the selected area (as shown in Figure 4a). It exhibits the presence of tungsten and sulfur elements over the selected area, confirming the successful formation of the WS_2_ composition. These results are well-matched and supported by the HRTEM and FFT results of the WS_2_. 

### 3.2. Acetone-Sensing Characteristics

The temperature and test gas concentration mainly affect the gas-sensing properties of the sensor. Therefore, it is necessary to find the optimum working temperature for a specific gas concentration of the chemical gas sensors. In light of this, we investigated the acetone-sensing properties of the WS_2_ nanorod sensor at 25 °C–100 °C for 5 ppm, 10 ppm, and 15 ppm concentrations. The desired acetone concentration of the WS_2_ nanorod sensor was evaluated with the help of Equation (1). In addition, the acetone gas sensitivity [*S* (%)] of the WS_2_ nanorod sensor was estimated using Equation (2) [50]:

(2) $$S\left(\%\right)=\frac{R_{a}-R_{acetone}}{R_{a}}\times 100$$

where *R_a_* and *R_acetone_* are the resistances measured under air and different acetone gas concentrations at different operating temperatures. 

**Figure 5:**
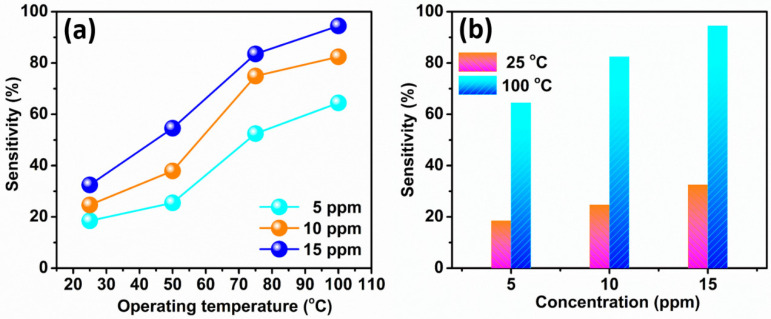
(**a**) Acetone sensitivity vs. temperature plots and (**b**) acetone sensitivity vs. concentration plots of the WS_2_ nanorod sensor.

Figure 5a shows the sensitivity vs. temperature plots of the WS_2_ nanorod sensor for 5, 10, and 15 ppm acetone concentrations. It is found that the sensitivity increases with the increasing acetone concentration and operating temperature of the WS_2_ nanorod sensor. The WS_2_ nanorod sensor shows the highest sensitivity of 94.5% for 15 ppm of acetone at 100 °C. However, the WS_2_ nanorod sensor reveals the lowest sensitivity of 18.5% for 5 ppm of acetone at 25 °C. The high sensitivity observed at 100 °C compared to the low sensitivity at 25 °C, 50 °C, and 75 °C for increasing concentrations may be attributable to the following reasons: (i) The high thermal energy at 100 °C (as compared to 25 °C) allows more thermally excited electrons to reach the conduction band, which can easily interact with the oxygen molecules to form the active site on the WS_2_ nanorod sensor surface; (ii) the large surface area of the 2D WS_2_ nanorod provides more interactions of oxygen molecules to form a large amount of active site on the sensor surface; (iii) the high electronic/ionic conductivity of WS_2_; (iv) high electronic and chemical responsiveness; and (v) rapid adsorption/desorption and extremely high diffusion of acetone molecules, leading to fast and outstanding sensitivity [51,52,53]. Figure 5b manifests the sensitivity vs. acetone concentration plots of the WS_2_ nanorod sensor at two operating temperatures of 25 °C and 100 °C. It is perceived that the WS_2_ nanorod sensor divulges low sensitivities of 18.5%, 24.7%, and 32.5% at an operating temperature of 25 °C for 5, 10, and 15 ppm of acetone, respectively. However, the WS_2_ nanorod sensor displays high sensitivities of 64.5%, 82.4%, and 94.5% at an operating temperature of 100 °C for 5, 10, and 15 ppm of acetone, respectively. It is also elucidated that the sensitivity increases with the acetone concentration at 25 °C and 100 °C. Many factors influence the sensitivity of the WS_2_ nanorod sensor due to the following reasons: (i) The high diffusion rate of gas molecules on the WS_2_ nanorod sensor surface due to the high concentration gradient of acetone molecules. The concentration gradient is proportional to the diffusion rate; therefore, acetone sensitivity increases with the acetone concentration at 25 °C and 100 °C. Furthermore, (ii) the acetone molecules formed a significant dipole moment due to the presence of the C-C=O group. It encourages the chemical adsorption/desorption and redox reaction capability of the WS_2_ nanorod sensor material [3,54].

**Figure 6:**
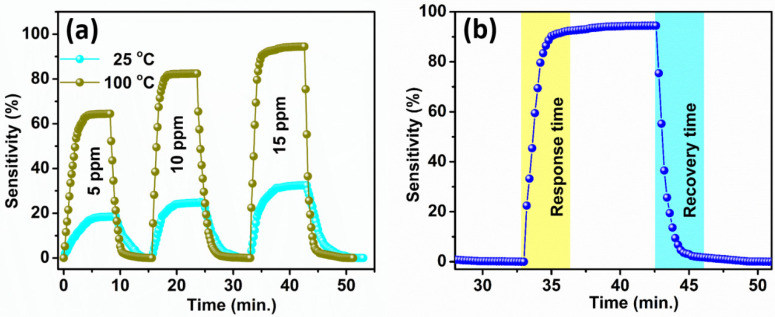
The WS_2_ nanorod sensor: (**a**) Transient characteristics at 25 °C and 100 °C for 5, 10, and 15 ppm acetone concentrations and (**b**) estimation of response and recovery time at 100 °C for 15 ppm of acetone.

Figure 6a shows the transient characteristic of the WS_2_ nanorod sensor at 25 °C and 100 °C for 5, 10, and 15 ppm of acetone. It shows a fast response and recovery at all acetone concentrations. It also exhibits that as the acetone concentration increases, the recovery and the response time decrease. This may be because of the enhanced chemisorption rate of acetone molecules on the WS_2_ nanorod sensor’s surface [55,56]. Figure 6b illustrates the response and recovery time plot of acetone molecules of the WS_2_ nanorod sensor. The response and recovery times were estimated when the WS_2_ nanorod sensor attained 90% of its maximum value and recovered 90% of its minimum value [57]. The WS_2_ nanorod sensor displays a quick response time of 3.02 min and a recovery time of 3.41 min at 100 °C for the 15 ppm concentration. This may be due to the high surface area of WS_2_ nanorods, rapid adsorption/desorption, and the chemisorption process. On the other hand, the reduction in the depletion layer and potential barrier height is due to more thermally excited electrons in the conduction band, leading to quick response and recovery [58,59].

**Figure 7:**
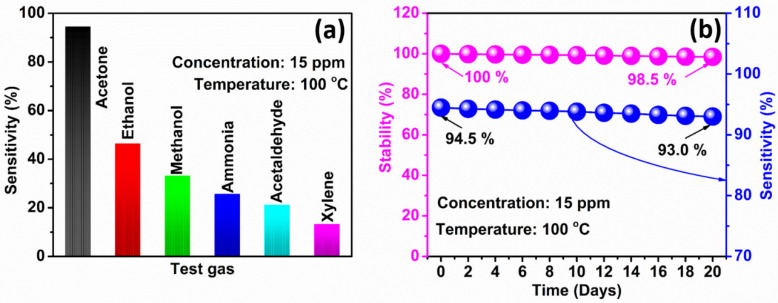
(**a**) Selectivity and (**b**) stability and corresponding sensitivity of the WS_2_ nanorod sensor.

Interestingly, the selectivity of the gas sensor plays a vital role in distinguishing the specific gas from various other gases. Here we investigated the selectivity of the WS_2_ nanorod sensor to six gases, including acetone. Figure 7a shows the sensitivity vs. test gas plots of the WS_2_ nanorod sensor at 100 °C for 15 ppm concentrations of various gas to disclose the selectivity behavior. It is detected that the WS_2_ nanorod sensor reveals the maximum sensitivity to acetone (94.5%) compared to the other gas, such as ethanol (46.4%), methanol (33.2%), ammonia (25.7%), acetaldehyde (21.2%), and xylene (13.2%) at 100 °C for 15 ppm concentrations. In addition, the selectivity coefficient (*C_s_*) to quantify the sensitivity of the WS_2_ nanorod sensor was calculated using the following Equation (3) [60].

(3) $$C_{s}=\frac{S_{acetone}}{S_{other\;gas}}\;$$

The estimated values of *C_s_* of the WS_2_ nanorod-based acetone sensor are 2.0 (ethanol), 2.9 (methanol), 3.7 (ammonia), 4.5 (acetaldehyde), and 7.1 (xylene). These *C_s_* values indicate that the sensitivity of the WS_2_ nanorod sensor to acetone is 7.1, 4.5, 3.7, 2.9, and 2.0 times higher than xylene, acetaldehyde, ammonia, methanol, and ethanol, respectively. Therefore, it is concluded that the WS_2_ nanorod sensor is most suitable for acetone detection compared to other tested gases at 100 °C with 15 ppm concentrations. The lowest unoccupied orbital energy has different values for different gases [61]. Figure 7b depicts the stability plot of the WS_2_ nanorod sensor for 20 days for 15 ppm concentrations at 100 °C. The sensitivity of the WS_2_ nanorod sensor slowly reduces with time from 94.5% (on the 1st day) to 93.0% (on the 20th day). It also exhibits the excellent stability of the WS_2_ nanorod sensor of 98.5% over twenty days for a 15 ppm acetone concentration at 100 °C. The high stability of the WS_2_ nanorod sensor may be due to the excellent electrical and thermal conductivity of WS_2_. On the other hand, the nanorod’s large surface area also provides high exposure to acetone molecules and rapid interactions with the adsorbed oxygen-active ions (O^−^), leading to excellent stability. 

In addition, a comparison between the various TMD-based acetone sensors has been discussed here with our observed results of the WS_2_ nanorod sensor. The MoS_2_-CuO nanocomposite sensor exhibited a high response of 16.21 for 10 ppm acetone at room temperature. It also showed a fast response of 61 s and recovery of 85 s [62]. The WS_2_/WO_3_ sensor has demonstrated a prolonged response time of 823 s and recovery time of 1093 s at 100 °C for 20 ppm acetone [42]. The decoration of Co_3_O_4_ on ZnS nanorods has been discussed regarding the acetone-sensing characteristics, which elucidated a high response of 1650% for 500 ppm acetone at room temperature under 2.2 mW cm^−2^ UV illumination [63]. A 2D SnS nanoflakes-based sensor displayed a high response of 1000%, a response time of ~35 s, and a recovery time of ~45 s at 100 °C for 10 ppm acetone [64]. SnS_2_-based sensors have demonstrated a high response of ~25, a response time of ~210 s, and a recovery time of ~600 s at 300 °C for 10 ppm acetone [65]. We found very limited reports on the WS_2_-based acetone sensor in the literature. Therefore, it is concluded that the WS_2_ nanorods could be a promising nanomaterial for an acetone sensor. 

### 3.3. Oxygen Active Site Formation and Acetone Molecule Detection Mechanism

The acetone recognition mechanism of the WS_2_ nanorod sensor essentially depends on the change in sensor resistance during gas sensing. The chemisorption reaction among the adsorbed active sites (O^−^) on the WS_2_ nanorod surface and acetone molecules determines the gas-sensing process [66]. It regulates the concentration of oxygen molecules with the WS_2_ nanorod sensor surface and modulates the sensor resistance [67]. Equations (4)–(6) represent the interaction reactions of atmospheric oxygen molecules and the creation of active sites (O^−^) on the WS_2_ nanorod surface at different operating temperatures [51,68,69].

(4) $$O_{2}\left(atmospheric\right)↔O_{2}\left(adsorbed\right)$$

(5) $$O_{2}\left(adsorbed\right)+e^{-}↔O_{2}^{-}\left(adsorbed\right)\;<\;100\;{}^{\circ}C$$

(6) $$O_{2}^{-}\left(adsorbed\right)+e^{-}↔2O^{-}\left(adsorbed\right)\;100\;{}^{\circ}C–300\;{}^{\circ}C$$

These active oxygen ions/sites are responsible for interacting with the acetone molecules. Therefore, the possible reaction between the adsorbed active sites (O^−^) and acetone molecules on the WS_2_ nanorod sensor surface is discussed in Equation (7) [59,70].

(7) $$CH_{3}COCH_{3}+8O^{-}↔3CO_{2}+3H_{2}O+8e^{-}$$

**Figure 8:**
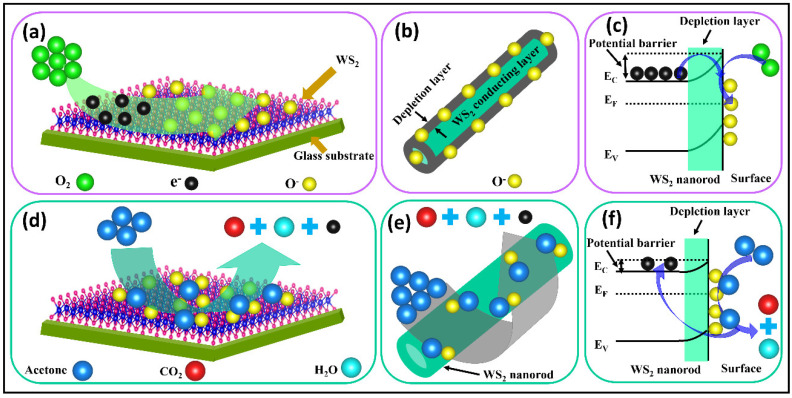
WS_2_ nanorod sensor: (**a**,**d**) Graphical illustration of adsorption of oxygen via electron interactions, formation of the active sites on the surface, and interaction of active sites with the acetone molecules; (**b**,**e**) visualization of a depletion layer formation around the WS_2_ nanorod by adsorbed active sites, and acetone-sensing reaction mechanism; (**c**,**f**) electronic band structure during active site formation and chemisorption of acetone molecules of the WS_2_ nanorod sensor.

Figure 8a–f show the schematic drawings of the oxygen adsorption reaction, depletion layer formation, the creation of a potential barrier, and reaction mechanisms of acetone molecules with the oxygen active sites (O^−^) on the WS_2_ nanorod surface. Figure 8a–c depict the schematic sketch of the creation of active sites on the WS_2_ nanorod sensor surface and depletion region in the electronic band structure. Firstly, atmospheric oxygen [*O*_2_(atmospheric)] was adsorbed on the WS_2_ nanorod sensor surface (*O*_2_(adsorbed)) using the process discussed in Equation (4). Further, it is expected that, below 100 °C, the adsorbed oxygen (*O*_2_(adsorbed)) interacts with the electrons in the conduction band and creates the active sites (O_2_^−^) on the WS_2_ surface, as discussed in Equation (5). After that, active sites (O_2_^−^) take more thermally excited electrons from the conduction band of the WS_2_ nanorod, finally creating the active sites (O^−^) on the WS_2_ nanorod sensor surface, as discussed in Equation (6). Figure 8b portrays a schematic view of the emergence of a depletion layer around the WS_2_ nanorod sensor surface during the adsorption process, which plays an intensive role in acetone sensing. Figure 8c describes the electronic band structure of the WS_2_ nanorod sensor following the various steps as discussed in Equations (4)–(6). It illustrates that the depletion layer and potential barrier are created during the adsorption and active site (O^−^) formation on the WS_2_ nanorod sensor. Similar reports have studied and discussed the exploration of the concept of the creation of active oxygen ions/sites in the literature [71,72,73]. Figure 8d reveals the graphical visualization of the interaction between acetone molecules and the active site (O^−^) on the WS_2_ nanorod sensor surface, following the process discussed in Equation (7), showing the liberation of CO_2_ gas, H_2_O, and electrons in the conduction band of the WS_2_ nanorod. Figure 8e discloses an illustration of the acetone-sensing mechanism (as discussed in Equation (7)) on the WS_2_ nanorod sensor surface and the release of carbon dioxide, water, and electrons. Figure 8f unveils the electronic band structure of the chemisorption of acetone molecules on the WS_2_ nanorod sensor (as discussed in Equation (7)). It is observed that the declining depletion region and the height of the potential barrier are created during the adsorption and creation of the active site (O^−^) on the WS_2_ nanorod surface. It also frees electrons in the conduction band of the WS_2_ nanorod sensor during the release of the carbon oxide and water molecules. The sensitivity of the acetone increases with increasing temperature and concentration due to the reduction in the depletion region and potential barrier heights, as schematically illustrated in Figure 8a–f, which supports the results discussed in Figure 5 and Figure 6. Similar reports of acetone-sensing mechanisms have been studied and discussed in the literature [73,74,75,76].

## 4. Conclusions

In conclusion, we studied an acetone gas-sensing application based on WS_2_ nanorods (NRs). The WS_2_ nanorod sensor shows the highest sensitivity of 94.5% at 100 °C for 15 ppm acetone. It also discloses the admirable selectivity of acetone compared to other gases, such as xylene, methanol, ammonia, acetaldehyde, and ethanol at 100 °C with a 15 ppm concentration. Further, it demonstrates fantastic stability over 20 days at 100 °C for a 15 ppm concentration. Consequently, it is concluded that the WS_2_ nanorod can offer a new choice for fabricating reliable, low-cost, environmentally friendly acetone sensors for observing workplace safety.
